# A longer life at the top of Mount Olympus?

**DOI:** 10.1007/s12471-016-0894-7

**Published:** 2016-08-25

**Authors:** E. E. van der Wall

**Affiliations:** Holland Heart House, Netherlands Society of Cardiology, Utrecht, The Netherlands

At the time of this publication the 2016 Olympic Games in Rio de Janeiro, Brazil (Rio 2016) are long gone and the mindset of most people is already directed to the 2020 Summer Olympics in Tokyo, Japan. Rio 2016 hosted almost 11.000 athletes from 206 National Olympic Committees (NOCs). With 306 sets of medals, the games featured 42 Olympic sports, including rugby and golf. Paradoxically, as Rio became the first South American city in the Southern hemisphere to host the Summer Olympics, the Summer Games were held during the winter season of the host country. The Netherlands were represented by 242 participants, engaging in 26 different Olympic sports. In the ensuing Paralympic Games, 114 athletes competed for the Netherlands; for both Games this was a top score in number of attendees.

An intriguing question is always whether Olympic athletes live longer than the general population [1, 2]. Along those lines, several studies have addressed longevity in former Olympic athletes [3–7].

An interesting Dutch study by Zwiers et al. from 2012 examined data on 9989 individuals who competed in Olympic Games between 1896 and 1936 [3]. Among former Olympic athletes, engagement in disciplines with high intensity exercise did not bring a survival benefit compared with disciplines with low intensity exercise. Those who engaged in disciplines with high levels of physical contact even had a higher mortality than other Olympians later in life. However, the authors did not compare their findings with matched controls. In addition, the investigators used data from athletes who had participated in the Olympic Games between 1896 and 1936, so outcomes reflect consequences of exercise programs that were the common practice before World War II, and which have of course changed substantially and dramatically over the past 80 years.

Three more recent studies have shown significant beneficial effects on longevity in Olympic athletes [4–6]. Published in 2015, Clark and colleagues analysed data on 15,174 Olympic medallists from nine countries who had enjoyed success in the Olympic Games [4]. The athletes had participated in at least one of the Olympic Games between 1896 and 2010. The study found that more medallists than matched controls in the general population were alive 30 years after winning. This resulted in a relative survival advantage of 8 % compared with matched controls, translating into 2.8 additional years of life. Gold, silver, and bronze medallists each showed similar survival advantages.

A study from France, also published in 2015, examined 2403 elite athletes (601 women and 1802 men) with the aim to measure overall and disease-specific mortality of French female and male Olympians compared with the French general population [5]. It was hypothesised that Olympians, both women and men, have lower mortality rates than the general population. The Olympians participated in Summer or Winter Olympic Games from 1948 to 2010. The authors showed that the overall mortality in Olympians compared with that of their compatriots was 51 % lower among women and 49 % lower among men, indicating that the survival of Olympic athletes is significantly superior to that of the French general population. This study corroborates their previous findings in 203 male French Olympic rowers, who participated in at least one of the Olympic Games from 1912–2012, showing a significant benefit of lower overall mortality compared with the French general population [6]. Among rowers’ main causes of death, cardiovascular diseases are reduced in relation to their compatriots.

A more recent study from Poland, published this year, investigated survival data of male elite Polish athletes who participated in the Olympic Games from the years 1924 to 2010 [7]. Deaths occurring before the end of World War II were excluded for reliable estimates. A total of 1273 male elite athletes were preassigned to two categorical birth cohorts: Cohort I from years 1890–1919, and Cohort II from years 1920–1959. The main findings were that 1) in Cohort I, for every threefold reduction in mortality risk, the rate of ageing decelerated by 1 %; 2) socioeconomic transitions and interventions contributed to a reduction in mortality risk of 29 % for the general population and 50 % for Olympic athletes; and 3) there was an optimum benefit gained for reducing the rate of ageing from competitive sports in both cohorts. It was concluded that intensive physical training during youth should be considered an important factor to improve ageing and mortality risk parameters.

Recent evidence clearly shows that Olympic athletes live longer than the general population irrespective of country, gender, sport or medal. Potential explanations include genetic factors, physical activity, healthy lifestyle, and the wealth and status that come with international sporting glory [1, 8, 9]. In due time one will truly know whether our 242 Rio-2016 Dutch Olympians have lived longer and healthier that their non-Olympic compatriots [10, 11].
